# Illuminating extracellular nanovesicles through the spectroscopic lens: a mini review of cutting-edge insights and emerging applications

**DOI:** 10.3389/fbioe.2025.1592391

**Published:** 2025-05-09

**Authors:** Debarati Bhowmik, Mohamed T. Patel, Pola Goldberg Oppenheimer

**Affiliations:** ^1^ Advanced Nanomaterials Structures and Applications Laboratories, School of Chemical Engineering, College of Engineering and Physical Sciences, University of Birmingham, Birmingham, United Kingdom; ^2^ Healthcare Technologies Institute, Institute of Translational Medicine, Birmingham, United Kingdom; ^3^ Cavendish Laboratory, Department of Physics, University of Cambridge, Cambridge, United Kingdom

**Keywords:** extracellular vesicles, Raman spectroscopy, Raman imaging, disease diagnostics, SERS (surface enhanced Raman scattering)

## Abstract

Extracellular vesicles (EVs) are cell-derived particles that facilitate intercellular communication by carrying bioactive molecules like proteins and RNA, impacting both health and disease. Herein, the EVs' significance in physiological and pathological processes is reviewed, emphasising their potential as biomarkers for diseases including for instance, cancer, neurodegenerative disorders and cardiovascular conditions. The principles and applications of Raman spectroscopy (RS) - a powerful tool offering detailed molecular insights into EVs, are further examined. The non-destructive nature of this spectroscopic technique renders it invaluable for studying the molecular composition, purity and concentration of EVs. When EVs are isolated from accessible biofluids such as blood, urine or saliva, the overall process remains minimally invasive, enhancing its clinical applicability. The review highlights Raman spectroscopy’s role in identifying disease-related EVs, distinguishing subpopulations and enhancing our understanding of EVs in disease mechanisms and therapeutic applications.

## Overview snapshot of the extracellular vesicles

**FIGURE 1:**
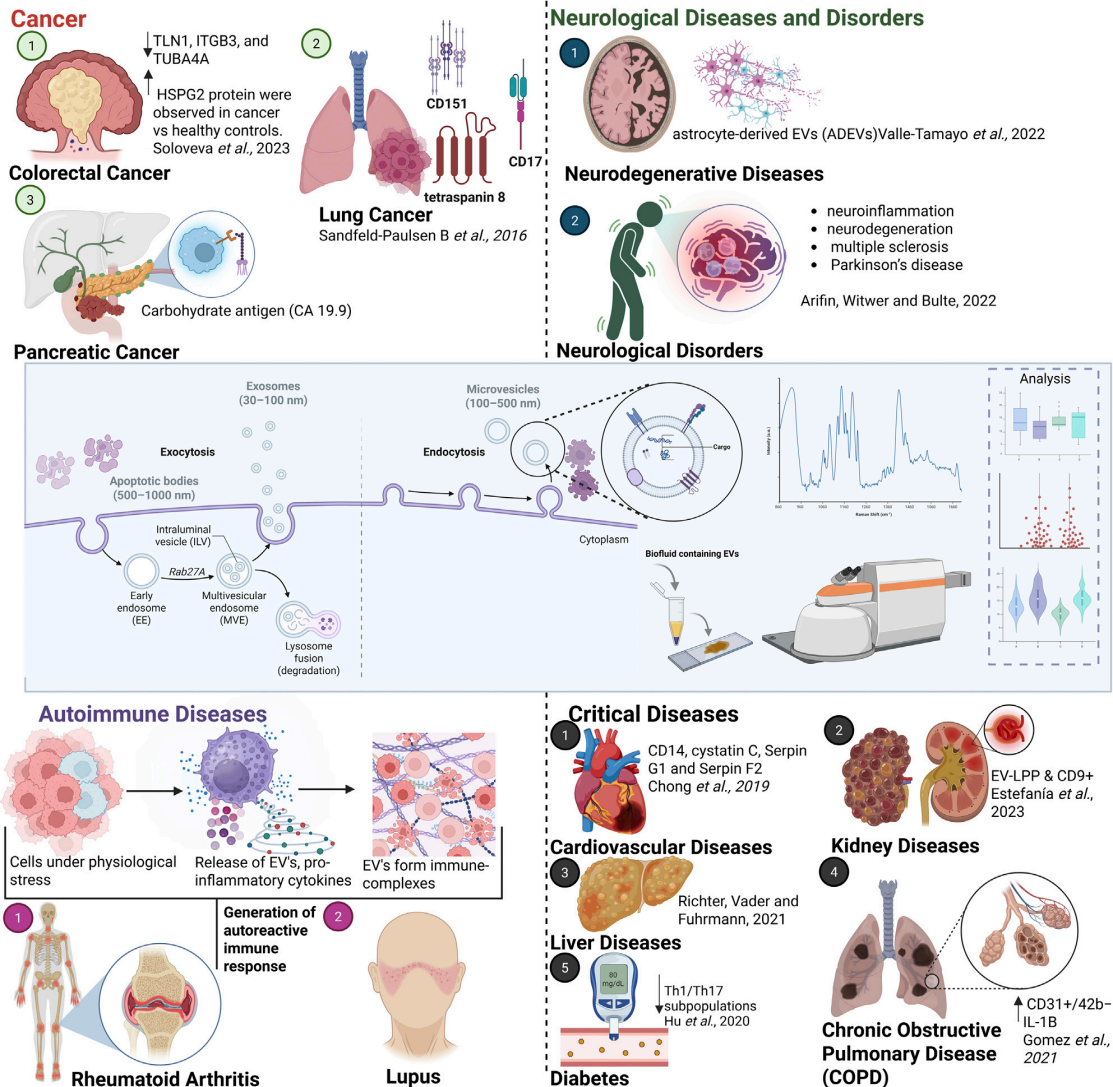
Schematic illustrating the importance of EVs in various diseases and conditions, including cancer (Sandfeld-Paulsen B et al.,2016), neurological (Arifin et al., 2022) and neurodegenerative disorders (Valle-Tamayo et al., 2022), autoimmune diseases, cardiovascular diseases (Chong et al., 2019), COPD (Gomez et al., 2022), liver (Richter et al., 2021) and kidney diseases (Estefanía et al., 2023) and diabetes (Hu et al., 2020). Created with Biorender (Bhowmik, 2025).

Extracellular vesicles are eukaryotic membrane-bound particles released by cells into the extracellular space, playing a pivotal role in both physiological and pathological processes by mediating intercellular communication through the transfer of proteins, lipids and nucleic acids. This ability to transfer bioactive molecules makes EVs significant in biological research and clinical applications (Muhsin-Sharafaldine and McLellan, 2018). EVs play a role in regulating diverse cellular processes, such as immune responses (Figure 1, Autoimmune Disorders) and tumour progression (Figure 1, Cancer). For instance, EVs derived from cancer cells can transfer oncogenic factors to healthy cells, promoting tumour growth and metastasis (Kalluri and McAndrews, 2023). Furthermore, EVs have emerged as valuable biomarkers for numerous diseases due to their capacity to reflect the molecular and physiological state of their originating cells. In colorectal cancer patients, the protein and RNA profiles of EVs can indicate disease presence and progression (Soloveva et al., 2023). Similarly, in neurodegenerative diseases like Alzheimer’s, EVs provide insights into disease mechanisms and aid in early diagnosis (Valle-Tamayo et al., 2022).

EVs are categorized into distinct types based on their size and biogenesis. Each type is generated differently and carries unique cargo, impacting its role as a biomarker and its detection by various analytical methods. Exosomes ranges from 30 to 150 nm in diameter, which are formed through the inward budding of endosomal membranes, resulting in the creation of multivesicular bodies, which fuse with the plasma membrane to release exosomes into the extracellular space. These are involved in intercellular signalling and immune modulation (Robbins and Morelli, 2014). Micro vesicles are larger than exosomes, with a size range of 100–1,000 nm and are generated directly from the budding of the plasma membrane. They participate in processes such as inflammation, coagulation, and cellular stress (Chong et al., 2019). Apoptotic bodies are the largest of EVs, typically larger than 1,000 nm apoptotic bodies are formed during apoptosis when cells break apart. They play a role in the clearance of apoptotic cells and debris (Muhsin-Sharafaldine and McLellan, 2018). Understanding the formation and functions of these vesicles is crucial for their application in diagnostics and therapeutics. EVs are also currently being explored for their potential in therapeutic applications, including drug delivery and gene therapy. EV’s natural ability to encapsulate and deliver therapeutic agents to target cells makes them promising candidates for novel treatments. Advances in EV engineering aim to enhance their targeting capabilities and therapeutic efficacy (Wang et al., 2023). Figure 1 (Neurological Diseases and Disorders, along with major conditions such as cardiovascular diseases, liver disorders, diabetes, kidney diseases and lung disorders) underscores the crucial role of EVs in a wide range of human pathological conditions. These diverse applications emphasise the crucial role EVs in both diagnostics and therapeutics, highlighting their potential to revolutionize disease monitoring and treatment.

As research advances, the ability to harness EVs for targeted drug delivery, early diagnosis and personalised therapy continues to expand, offering new hope for the management and cure of complex diseases. Central to this progress is the accurate determination of EV chemical composition, as their molecular cargo, including proteins, lipids and nucleic acids, not only reflects the physiological and pathological status of their parent cells but also defines their functional capabilities (Chiang and Chen, 2019; Kumar et al., 2024). This compositional specificity underpins their value as biomarkers for early disease detection and real-time monitoring, particularly in cancers (Fitzgerald et al., 2022), cardiovascular diseases (Fu et al., 2020), and neurodegenerative disorders (Zaborowski et al., 2015; Chen C. et al., 2024). Furthermore, a detailed understanding of EV content enables the development of advanced EV-based therapeutic strategies, including engineered drug delivery systems and regenerative medicine approaches, by ensuring targeted, efficient, and reproducible effects (Davidson et al., 2022). The ongoing development in EV engineering and molecular characterization thus paves the way for innovative medical interventions, potentially transforming the future of healthcare.

In this review, the applications of Raman spectroscopy and its sub-variations are overviewed for the characterisation of EVs whilst focusing on its unique capabilities for identifying and analysing the molecular composition of these vesicles as potential biomarkers of diseases. By comparing Raman spectroscopy with traditional EV characterisation techniques, the review highlights its advantages, such as label-free detection and non-destructive analysis and demonstrates the potential of Raman spectroscopy in identifying disease-specific EV signatures. The limitations of the technique, such as reduced sensitivity in detecting low biomarker concentrations or interference from autofluorescence, are also discussed. Additionally, recent advancements and emerging prospects, including technological innovations aimed at improving sensitivity and clinical relevance, are explored, providing a comprehensive resource for researchers and clinicians in biomedicine, diagnostics and pathology.

## Raman spectroscopy

Raman spectroscopy is a powerful, non-destructive vibrational spectroscopy technique based on the inelastic scattering of monochromatic light, typically from a laser. The phenomenon of light scattering can be explained using classical theory. According to classical theory, when a molecule is exposed to electromagnetic radiation-typically from a laser source, an oscillating electric field induces a time-dependent dipole moment in the molecule. The induced dipole moment *μ = α*E, where *α* is the polarizability of the molecule and E is the electric field of the incident radiation, governs the scattering of light (Jones et al., 2019). Most scattered photons are elastically scattered (Rayleigh scattering), retaining their energy, while a small fraction undergo inelastic scattering (Raman scattering), resulting in energy shifts that correspond to the vibrational modes of the molecule (Bhowmik et al., 2024). A complete Raman spectrum typically displays both Stokes and anti-Stokes scattering symmetrically about the Rayleigh line, though Stokes scattering is more commonly observed due to the higher population of molecules in the ground vibrational state. Raman spectroscopy thus provides a molecular fingerprint, enabling structural elucidation through the identification of specific vibrational modes (Banwell, 1972). However, conventional Raman spectroscopy suffers from low signal intensity, as only approximately one in every 10^6^–10^8^ photons undergoes Raman scattering. This inherent weakness is exacerbated by the presence of background fluorescence, especially prevalent in biological samples and interference from cosmic rays and solvent scattering, which can significantly reduce the signal-to-noise ratio (Fernández-Galiana et al., 2023).

To overcome these limitations, Surface-Enhanced Raman Scattering (SERS) has emerged as a powerful variant of Raman spectroscopy. SERS enhances the Raman signal by factors of 10^6^–10^8^ or more through the adsorption of analyte molecules onto nanostructured metal surfaces, commonly silver or gold, capable of supporting localized surface plasmon resonances (LSPRs) (Pilot et al., 2019; Schlücker, 2014). This enhancement arises from two primary mechanisms. Electromagnetic enhancement, which results from the amplification of the local electromagnetic field near the metal nanostructure due to plasmon excitation. And chemical enhancement, which involves charge transfer interactions between the analyte and the metal surface that increase the molecular polarizability (Patel and Goldberg Oppenheimer, 2025).

Unlike conventional Raman spectroscopy, where the signal originates from the bulk of the sample, SERS and tip enhanced Raman spectroscopy (TERS) is a surface-sensitive technique, making it particularly well-suited for trace-level detection and label-free biosensing. One of its key advantages is the ability to analyse samples with strong fluorescent backgrounds, as the enhanced Raman signals can effectively outcompete fluorescence emission (Pilot et al., 2019). TERS in comparison to SERS offers superior spatial resolution down to the nanometre scale, enhanced signal localisation, improved reproducibility, and greater surface sensitivity, making it particularly advantageous for nanoscale chemical imaging and single-molecule analysis (Yi et al., 2024). Similarly, UV-SERS, and UV-Resonance Raman Spectroscopy (UVRRS) is a powerful analytical technique that utilizes two laser excitation wavelengths: a resonant ultraviolet (UV) wavelength (typically between 200 and 514 nm) and a non-resonant wavelength (commonly 785–1,064 nm). This dual-wavelength approach enables selective enhancement of Raman signals through resonance with UV excitation. UVRRS is particularly effective for biological molecules containing aromatic rings and amide groups such as proteins and DNA, which often exhibit strong fluorescence under conventional Raman spectroscopy. By using UV excitation, UVRRS significantly reduces fluorescence interference and enhances the Raman signal, allowing for more sensitive and specific detection of these biomolecules (Bhowmik et al., 2024). Although this technique has not yet been reported in the context of EV diagnostics, it holds significant potential to be explored for this purpose. However, the performance of SERS is highly dependent on the quality and architecture of the substrate, and imperfections such as inhomogeneity, instability, and limited tunability can significantly affect the spectral response. Substrate reproducibility and specificity remain a major challenge in practical applications. The magnitude of SERS enhancement is influenced by several factors, including the size, shape, and morphology of the metallic nanostructures. The electromagnetic enhancement mechanism is approximately proportional to the fourth power of the local electric field intensity, yet in practice, most analyte molecules are not located at the “hot spots” where the field is maximally amplified, resulting in a lower overall enhancement. Nevertheless, SERS signal intensity remains highly dependent on the excitation wavelength, necessitating precise tuning to match the plasmonic resonance of the substrate for optimal performance (Chang et al., 2025). Combined with the higher costs of fabricating robust, reproducible substrates, these technical constraints must be carefully managed to ensure reliable and quantitative application of SERS, particularly in sensitive domains such as extracellular vesicle (EV) diagnostics.

We discuss the potential applications of Raman spectroscopy beyond the laboratory, showcasing the technique’s versatility. We further foresee significant expansion in the use of SERS, a sub-technique of Raman spectroscopy, including the adoption of UV SERS as well as the TERS in biological diagnostics, sensing and imaging. Despite its broad potential, Raman spectroscopy still faces challenges (Jones et al., 2019). The spontaneous Raman intensity is weak and can be easily interfered with by other signals such as fluorescence, cosmic rays from charged-couple device (CCD) and scattering from solvents in practical applications (Fernández-Galiana et al., 2023). Biological samples also often have a strong fluorescence background, which can reduce the signal-to-noise ratio (SNR) of Raman spectra. However, this can be mitigated by using techniques such as SERS, TERS, resonance Raman spectroscopy, stimulated Raman spectroscopy (SRS) and coherent anti-Stokes Raman scattering (CARS) (Jones et al., 2019).

## Advancements in biomedical applications

In recent years, SERS has been significantly advancing new biosensing technologies. However, despite its potential, the progress towards becoming a widely adopted clinical diagnostic tool has been hindered, largely due to its limited tissue penetration depth. Thereby, to overcome this issue, diagnosis have moved towards liquid biopsies and further, EV’s as biomarkers for disease diagnostics. Nevertheless, SERS has demonstrated clear advantages over other imaging techniques such as fluorescence imaging and magnetic resonance imaging (MRI) in certain clinical areas (Li et al., 2023). The future of single-cell imaging is likely to be dominated by SERS-based technology due to its superior brightness, high sensitivity and resistance to photobleaching. Additionally, SERS provides better resolution at the micrometre scale compared to MRI, which typically offers resolution in millimetre range. For instance, MRI requires the presence of around 100,000 cancer cells in a tumour for detection, which may be too late for aggressive cancers. The advantages of SERS in biosensing are expected to be harnessed further, paving the way for its development into a clinically viable diagnostic technology (Liu et al., 2022).

Early and accurate disease detection is crucial for effective treatment, monitoring treatment progress, slowing disease progression and reducing mortality (Fitzgerald, et al., 2022). For example, in colorectal cancer (CRC), early-stage diagnosis can lead to a 5-year survival rate of up to 90%. However, only 39% of CRC patients are diagnosed early, often due to missed lesions and underutilization of screening. Similar trends are seen in other diseases, including infectious (Baker, et al., 2022) and neurodegenerative conditions. Consequently, there is a constant demand for innovative, reliable detection technologies with high specificity and sensitivity (Dave, V. P., et al., 2019). Optical diagnostic techniques, such as Raman spectroscopy, offer several advantages, including objectivity, speed and cost-effectiveness (Kim et al., 2020). Raman spectroscopy, which detects biochemical components in biological tissues, has garnered attention for clinical applications due to its robust performance, ease of use, specificity and sensitivity in detecting and grading lesion tissues (Butler et al., 2016). This spectroscopic technique identifies the composition of multicomponent substances based on the characteristic molecular vibrations within the sample (Qi et al., 2024). Coherent anti-Stokes Raman scattering (CARS) has been successfully employed by Enciso-Martinez et al. (2019) to optically trap and analyse individual EVs, enabling the distinction of tumour-derived EVs (tdEVs) from other particles and EVs present in body fluids. Building on this, Gong et al. (2019) proposed the use of Higher-Order Coherent Anti-Stokes Raman Scattering (HO-CARS) microscopy, a refined version of CARS, to achieve label-free, high-contrast nanoscale imaging.

Human tissues, composed of proteins, nucleic acids and lipids, exhibit distinct peaks in the Raman spectra, providing valuable information. For example, Stepanenko et al., 2023, have explored TERS for label-free nanoscale characterisation of EVs and their isolated membranes derived from red blood cells (RBCs), enabling single-molecule level detection of individual amino acids, as well as distinct protein and lipid membrane components. In lesioned tissues, molecular composition and structure change as the disease progresses, allowing for detection at the cellular and molecular levels. Spectroscopic techniques can accurately and sensitively capture these molecular fingerprints, offering a feasible method for minimally invasive disease detection (Zhang et al., 2023). Raman spectroscopy further shows promise for diagnosing progressive diseases such as cancer, precancerous lesions (Qi et al., 2024), infectious diseases (e.g., COVID-19, dysentery, dengue fever, epidemic hepatitis) (Jadhav et al., 2021) and metabolic diseases like osteoporosis and diabetes (Chen K. et al., 2024). Although tip-enhanced Raman spectroscopy (TERS) exhibits impressive *in vitro* performance, it’s *in vivo* application remains constrained by nanometre-scale probe alignment requirements, limited tissue accessibility, signal attenuation in scattering biological media, and the challenge of sustaining stable tip-enhancement in dynamic environments. To address these limitations, the development of miniaturised, fibre-coupled AFM and integrated micro-endoscopy probes equipped with plasmonic tips have been proposed, enabling direct sampling of EV from rich biofluids such as blood and cerebrospinal fluid for real-time, label-free molecular analysis. Whereas, Ultraviolet surface-enhanced Raman scattering (UV-SERS) similarly offers strong resonance enhancement for biomolecular chromophores but is hindered *in vivo* by UV-induced photodamage and shallow optical penetration. Thus, Implementing near-UV excitation (300–350 nm) to balance resonance enhancement with biocompatibility, or integrating on-chip fluid biopsy platforms featuring UV-active plasmonic substrates, may effectively mitigate these challenges (Schlücker, 2014; Zhang et al., 2020). SERS is undoubtedly a promising technique for diagnostic purposes and for use as point-of-care devices. However, realizing its full impact requires overcoming key challenges through further advancements in substrate fabrication, refining assay techniques, and integrating platforms effectively whilst ensuring these are cost-effective. The discussion has shown that Raman spectroscopy allows for the continuous and highly sensitive detection and quantification of various biomarkers and end-products of disease states, rendering it an excellent option in the early-stage diagnosis and treatment of various diseases that are cause health concerns. The advances in the development of the various adjacent, novel techniques, e.g., UV-SERS, TERS and SERS will remain indispensable due to these showing a great promise for *in-vitro* and *in-vivo* disease detection. Thus, emphasising their broad applied uses to detect and characterise EVs for disease diagnostics.

## Importance of integrating Raman spectroscopy in EV research

The conventional methodologies for examining biochemical composition of EVs are typically characterised using a range of analytical techniques which provide detailed insights into their molecular makeup. Western blotting is commonly employed to detect specific proteins associated with EVs, such as tetraspanins (CD9, CD63, CD81), heat shock proteins and other cellular markers, helping to confirm the presence and origin of the vesicles. Flow cytometry, despite requiring specialized equipment due to the small size of EVs, allows for the analysis of surface markers on individual vesicles, making it useful for assessing the heterogeneity within EV populations. Mass spectrometry (MS) is a powerful tool for performing detailed proteomic analysis, enabling the identification and quantification of proteins, lipids and other biomolecules within EVs.

Furthermore, advanced MS techniques, such as lipidomics and metabolomics, are used to analyse the lipid and metabolite content of EVs, providing deeper insights into their biochemical profile and functional roles. Recently, optical Raman spectroscopy has been emerging as a new powerful analytical tool for EV research, offering detailed insights into their molecular composition and functionality. Its label-free nature avoids biases from dyes or antibodies, offering clearer disease-relevant information.

Raman spectroscopy’s ability to characterize EVs in a non-destructive manner makes it invaluable for studying these small, membrane-bound particles (Zini et al., 2022). When EVs are isolated from body fluids such as blood or urine, the combined process is minimally invasive, supporting its potential use in clinical diagnostics. EVs contain a diverse molecular cargo including lipids, proteins, nucleic acids and sugars, that can serve as biomarkers for various diseases. Once absorbed by recipient cells, EVs can initiate intercellular signalling and influence intracellular metabolic processes (Abels and Breakefield, 2016). It also plays a crucial role in assessing the purity and concentration of EV samples. Zini et al. (2022) highlighted its utility, alongside an Attenuated Total Reflectance-Fourier Transform Infrared (ATR-FTIR), in determining lipid-to-protein ratios (Li/Pr), which are indicative of EV purity. The technique’s capacity to analyse small sample volumes further enables a practical purity assessment. Another study demonstrated the effectiveness of Raman spectroscopy for bulk characterisation of EVs from various sources, confirming its ability to differentiate EVs from bone marrow and adipose tissue. This validation underscores Raman spectroscopy’s role in purity and concentration estimation (Gualerzi et al., 2017).

## Identification and characterization of EVs from various biological sources

**FIGURE 2:**
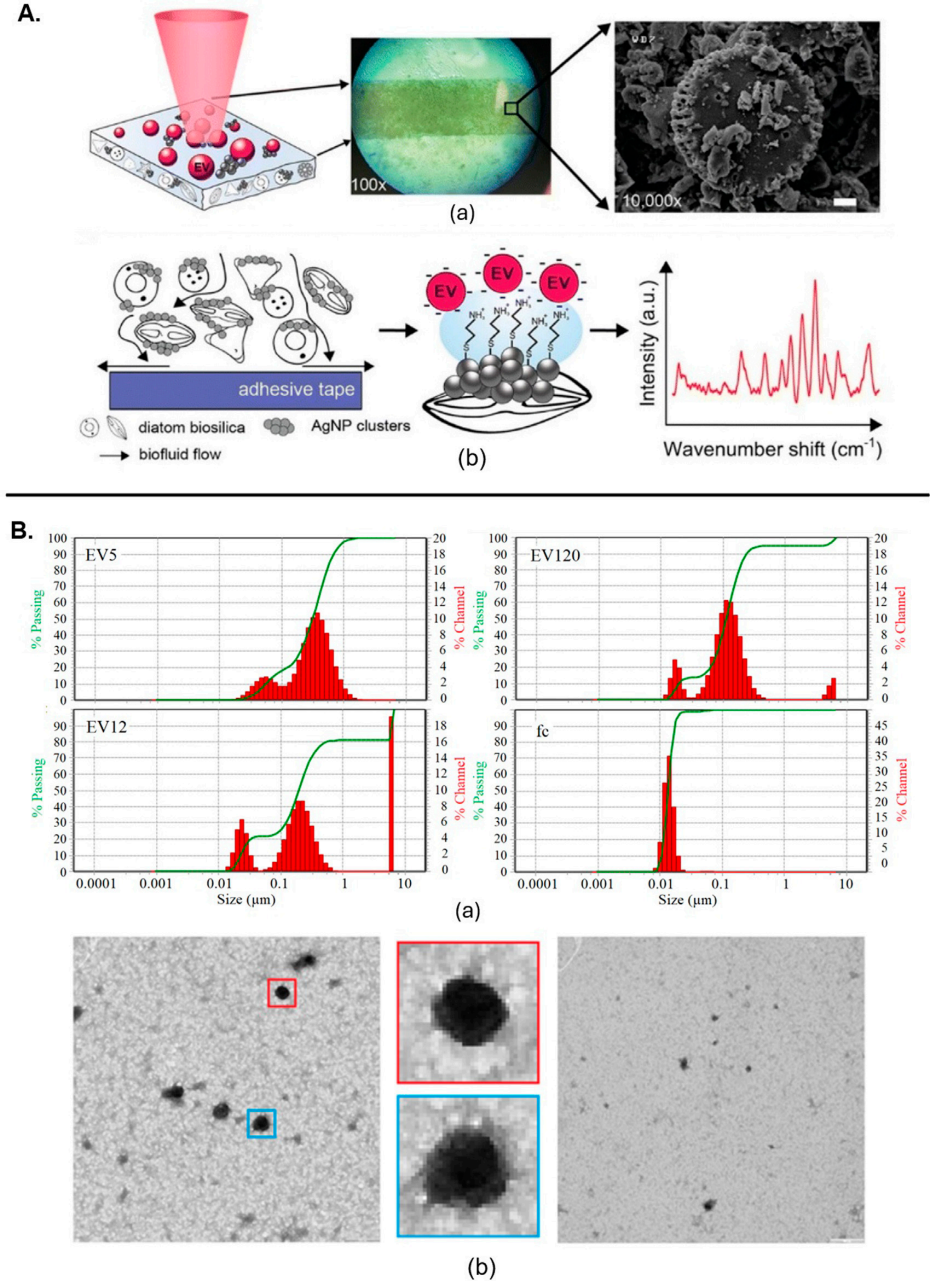
**(A)** SERS substrate irradiated by laser light initiates (a) Raman scattering. (b) The substrate facilitates the transport of EVs near AgNP clusters on the silicate scaffold (Zheng et al., 2022). **(B)** Characterization of EVs from citrate plasma samples: (a) Particle size distribution by dynamic light scattering. (b) TEM of EV120-enriched fraction, with enlarged views showing typical EV structures (Osei et al., 2021). All figures are reprinted with permission.

EVs derived from multiple cell lines have been shown to be successfully classified using a combination of SERS and machine learning algorithms. This method effectively distinguished cancer-derived EVs from those originating from healthy cells, highlighting RS’s potential for identifying disease-related EVs (Parlatan et al., 2023). Similarly, it was also used to differentiate between EVs from bovine placenta and peripheral blood mononuclear cells, identifying specific Raman peaks associated with collagen and cholesterol displaying its ability to distinguish EVs based on their biochemical content (Zhang et al., 2020). Another study introduced time-gated Raman spectroscopy (TG-RS) and surface-enhanced time-gated Raman spectroscopy (TG-SERS), offering improved sensitivity and resolution for studying EVs under various conditions, such as hypoxia as shown in Figure 2A (Zheng et al., 2022). The integration of quasi-bound states in the continuum (quasi-BICs) meta-surfaces with RS has also improved the handling and analysis of numerous small EVs simultaneously, addressing the limitations of traditional optical trapping methods (Hasan and Hellesø, 2023).

## Comparative analysis of EV subpopulations

**FIGURE 3:**
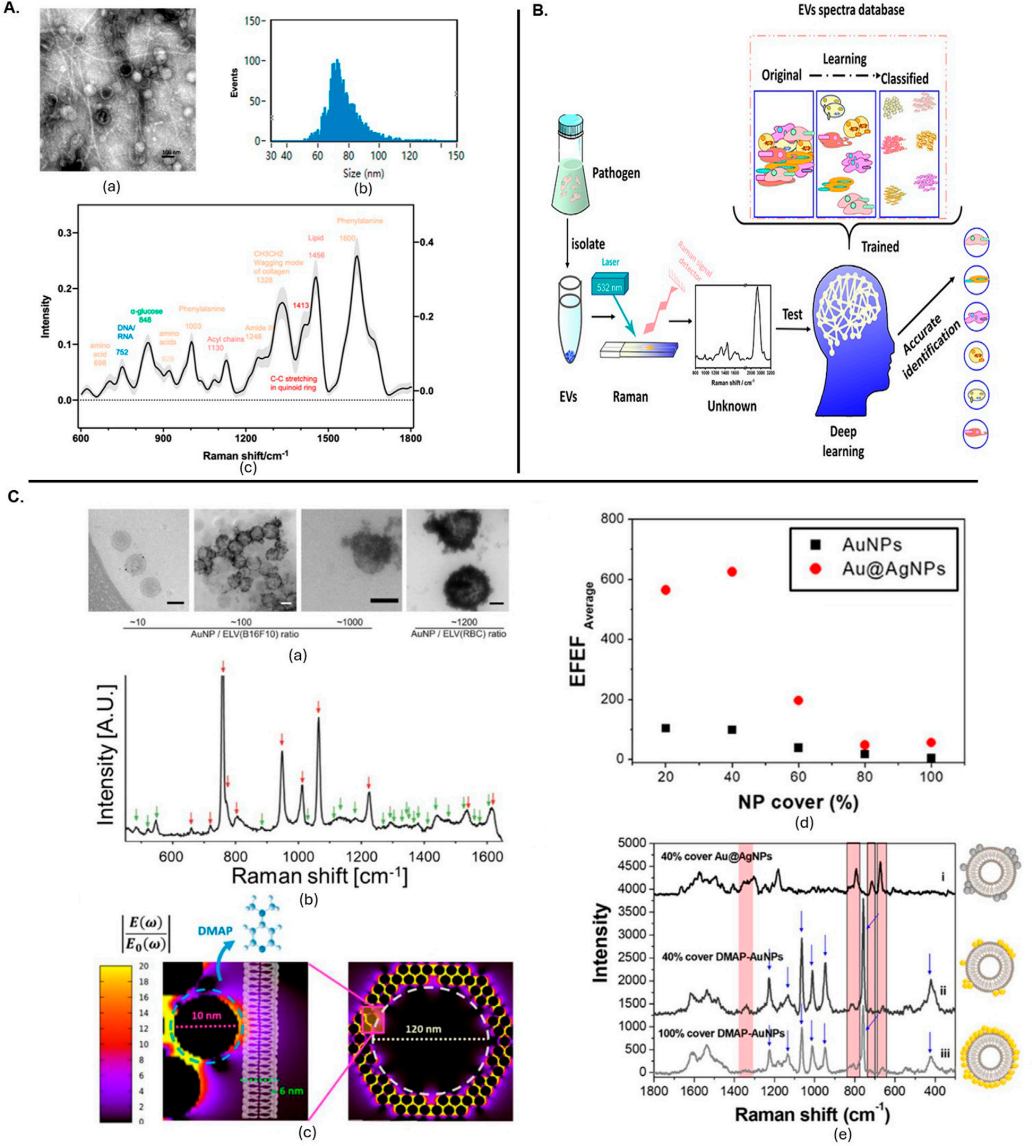
**(A)** The EVs released from *Pseudomonas aeruginosa* PAO1 were characterized using several techniques. (a) Transmission electron microscopy images showed the morphology of these EVs, with a scale bar of 100 nm. (b) The particle size distribution was analysed by nanoflow cytometry, revealing sizes ranging from 68 to 155 nm using standard-sized particles as references. (c) Raman spectroscopy provided the spectral profile of the EVs, with the mean and standard deviation represented by a solid line and light shading, respectively, with different colours indicating various substance types (Qin et al., 2023). **(B)** Deep learning-enabled Raman spectroscopic identification of pathogen-derived EVs and their biogenesis (Qin et al., 2022). **(C).** Positively charged particle-based EV detection. (a) TEM images of DMAP-AuNPs-coated EVs at different ratios, scale bars 100 nm. (b) SERS measurements of DMAP-AuNP coated EVs, with DMAP and EV peaks indicated. (c) EM field enhancement with a zoom-in of a single DMAP-AuNP on a vesicle surface. (d) Relation between EV coverage ratio and EFEF before and after Ag shell formation. (e) SERS characterization of EVs with Ag shell-AuNPs or DMAP-AuNPs, showing various coverage percentages (Shin et al., 2020). All figures are reprinted with permission.

RS is also valuable for distinguishing between EV subpopulations. A combination of Raman spectroscopy with gas chromatography-time-of-flight mass spectrometry (GCxGC-TOFMS) was used to analyse exomere- and exosome-sized EVs. This comprehensive approach revealed differences in biochemical compositions between these subpopulations, highlighting its utility in detailed EV analysis (Liangsupree et al., 2022). The use of both spontaneous and SERS to analyse EV-enriched fractions from plasma samples of prostate cancer patients enabled effective discrimination between cancer-related and control samples with its particle size distribution and Fc fraction (Figure 2B) (Osei et al., 2021). Further, a multi-modal analysis platform combining Raman spectroscopy with scanning electron microscopy and atomic force microscopy allowed for the specific capture and detailed analysis of tumour-derived EVs on antibody-functionalized substrates. This approach provided complementary data on size distribution, chemical fingerprints and spatial correlation (Beekman et al., 2019). RS, when combined with multi-omics, has been used to analyse EVs from *Pseudomonas aeruginosa* PAO1, revealing insights into macrophage internalization and metabolic alterations as shown in schematic (Figure 3A) (Qin et al., 2023). SERS combined with a deep learning model demonstrated high accuracy in classifying pathogen-derived EVs, including Gram classification and strain identification. This study uncovered spectral markers for distinguishing between different bacterial origins as shown in Figure 3B (Qin et al., 2022).

Zheng et al. (2022) reviewed recent progress in EV detection, covering both direct label-SERS and indirect labelling strategies. The review highlighted the advantages of specific SERS-related substrate fabrication and nanoprobe assembly for EV characterization. Similarly, another study discussed label-free SERS approaches for identifying disease-related EV patterns, emphasizing the use of plasmonic nanostructures and signal analysis strategies to detect and interpret complex EV spectra, and addressing challenges and prospects in EV research. Figure 3C shows the positively charged particle-based EV detection with TEM imaging and SERS spectra obtained from them (Shin et al., 2020). RS ability to distinguish between EVs and conditioned medium (CM) from different cell types, revealing insights into the reproducibility of EV isolation and the role of soluble factors in CM (Carlomagno et al., 2021).

## Conclusions and future prospects

The integration of Raman spectroscopy into EV research has significantly deepened our understanding of their molecular composition, functionality and potential as disease biomarkers. Its non-destructive characterization of EVs has proven invaluable for diagnostics, therapeutic monitoring and drug delivery. When EVs are obtained from accessible biofluids, the overall process can be achieved with minimal invasiveness, making it well-suited for routine clinical use. Raman spectroscopy has demonstrated effectiveness in identifying disease-associated EVs, differentiating between subpopulations and providing insights into pathological processes.

Looking ahead, the future of Raman spectroscopy in EV research is highly promising. Emerging techniques such as time-gated Raman, surface-enhanced Raman, and the incorporation of machine learning are expected to greatly enhance sensitivity, resolution and overall analytical power. These advancements will enable more precise and comprehensive characterization of EVs, allowing for the identification of specific disease markers and the development of targeted therapies. However, a key-limitation in the diagnostic application of vibrational spectroscopy, including Raman and its enhanced variants, lies in the absence of universally recognised protocols governing measurement practices, spectral pre-processing techniques and downstream data analysis approaches, either as univariate or multivariate. This lack of methodological standardisation presents significant challenges in ensuring reproducibility, comparability and translational validity across different studies and clinical settings. Variations in acquisition parameters, such as laser wavelength, exposure time and spectral resolution as well as inconsistent application of baseline correction, normalisation or noise-reduction strategies, can lead to divergent interpretations of otherwise similar biological samples. Recognising this gap, recent efforts have been made to establish best practices and consensus-driven guidelines. Morais et al. (2020) offer an extensive tutorial focused on multivariate classification approaches in vibrational spectroscopy of biological samples, outlining preprocessing steps such as Savitzky-Golay filtering, vector normalization and PCA-based dimensionality reduction. This work further highlights model validation techniques which reduce overfitting and enhance diagnostic robustness. Further, Butler et al. (2016) provide a comprehensive framework for the characterisation of biological materials via Raman spectroscopy, with particular attention to instrumentation, sampling techniques and data interpretation in complex biological matrices. More recently, Traynor et al. (2021) have detailed a Raman spectral cytopathology protocol optimised for cancer diagnostics, covering specimen preparation, spectral acquisition and a complete data analysis pipeline including quality control checks as well as classification strategies using multivariate models such as linear discriminant analysis and partial least squares discriminant analysis (PLS-DA). The principles outlined in these studies are increasingly relevant to the analysis of EVs, where subtle biochemical differences must be robustly identified across heterogeneous populations and biofluids. Given the nanoscale and compositional variability of EVs, standardisation becomes even more critical when seeking spectral biomarkers for disease states. Integrating such standardised workflows, particularly those emphasising reproducibility and spectral integrity, into EV-focused vibrational spectroscopy studies could greatly enhance cross-study comparability and clinical confidence. Future work should prioritise adapting and validating these proposed methodologies in EV-specific contexts, incorporating them into diagnostic pipelines and where possible, establishing inter-laboratory benchmark datasets. In parallel, the development of open-access spectral databases and community-endorsed reporting standards will be pivotal in promoting transparency and advancing the field toward clinical translation.

Moreover, the combination of Raman spectroscopy with other analytical platforms such as flow cytometry, gas chromatography, time-of-flight mass spectrometry and multi-omics approaches holds substantial promise. These multimodal strategies can offer a more holistic and integrated view of the biochemical complexity of EVs, enabling the development of next-generation diagnostic tools and personalised therapeutic interventions. It is also crucial to acknowledge that different EV isolation techniques can significantly influence sample purity and spectral profiles, underscoring the need for continued research into standardisation and reproducibility. Furthermore, integrating Raman spectroscopy with microfluidic and lab-on-a-chip systems as well as developing real-time, high-throughput and multiplexed analytical workflows, represents a vital direction for future innovation. As these advancements converge, Raman-based EV diagnostics are poised to redefine disease monitoring, facilitate early, precise diagnosis and open new avenues in targeted therapy. Collectively, these developments signal a transformative shift toward more accessible, accurate and personalised healthcare solutions.
